# Learning from magnetotactic bacteria: *mms6* protects stem cells from oxidative damage

**DOI:** 10.3389/fncel.2022.1075640

**Published:** 2022-11-23

**Authors:** Nai-Li Wei, Wenjing Xu, Hai-Liang Tang, Qiang Xie, Yuting Zhai, Jian Chen, Xiao-Yong Zhang, Jian-Hong Zhu

**Affiliations:** ^1^Department of Neurosurgery, The First Affiliated Hospital of Shantou University Medical College, Shantou, Guangdong, China; ^2^State Key Laboratory for Medical Neurobiology, Department of Neurosurgery, Institutes of Brain Science, Fudan University Huashan Hospital, Shanghai Medical College-Fudan University, Shanghai, China; ^3^Institute of Science and Technology for Brain-Inspired Intelligence, Fudan University, Shanghai, China; ^4^MOE Key Laboratory of Computational Neuroscience and Brain-Inspired Intelligence, MOE Frontiers Center for Brain Science, Fudan University, Shanghai, China; ^5^Department of Neurosurgery, Fudan University Zhongshan Hospital, Shanghai Medical College of Fudan University, Shanghai, China

**Keywords:** stem cells, reactive oxygen species, *mms6*, antioxidant, mitochondrial function

## Abstract

Oxidative damage generally exists in stroke and impairs stem cells’ survival; however, the problem is difficult to treat. In order to help stem cells to resist this damage, we inserted a magnetotactic bacteria (MB) gene, *mms6*, into the neural stem cell genome by lentiviral transfection. It was found that the transfection of *mms6* significantly improved the survival rate of stem cells in the condition of iron overload but not hypoxia. The bioenergetic profile also revealed that iron overloading weakened the mitochondrial respiration and spare respiration capacity of stem cells, but that these were enhanced after the expression of *mms6*. Additionally, Western blotting (WB) data revealed that *mms6* upregulated the expression of glutathione peroxidase (GPX4), which protected stem cells from oxidative damage and ferroptosis. In order to determine the possible mechanisms, we analyzed the interactions between the MMS6 protein, Fe2+, and GPX4 via analog computation. The predicted models found that the MMS6 protein had a direct chelating site in the region of M6A with divalent iron; it also had weak binding with GPX4. Taken together, the magnetotactic bacterial gene *mms6* protected stem cells from oxidative damage via binding with Fe2+, which could help them adapt to the microenvironment of stroke.

## Introduction

Stem cell replacement therapy is frequently applied to treat stroke, but, in this disease, oxidative damage is often induced by pathological factors, such as hypoxia and iron overloading (Duce et al., 2010; Jiang et al., 2019). Oxidative damage seriously affects the fate of stem cells (Wilson et al., 2018). Moreover, the relatively hypoxic microenvironment after stem cell transplantation will lead to hypoxia and produce excessive reactive oxygen species (ROS) (Jiang et al., 2019), thus affecting the survival of stem cells. Recently, increasing studies proved that iron overload following up with ischemic or hemorrhagic stroke can produce detrimental ROS and brain injury and neuronal ferroptosis (Wan et al., 2019). The iron overloading usually occurs at the subacute and chronic phase of stroke following up with acute hypoxia, cellular excitotoxicity, and neuroinflammation (Qin et al., 2022). In order to treat stroke, stem cell transplantation is recommended to implement at this phase (Boese et al., 2018). In term of pathogenic injury process, oxidative damage induced by iron overloading may be a major hinder on survival rate of stem cells. To solve this problem, some researchers have employed drug carriers to reduce the ROS levels in stem cells (Gomes et al., 2019; Jiang et al., 2019), which improves the survival rate of stem cells. Nonetheless, these methods cannot solve the problem of oxidative damage in the host microenvironment. Notably, the problem of host environments after transplantation needs to be overcome for the long-term survival of stem cells.

Magnetotactic bacteria represent a kind of special archaea that grow in extremely iron-rich and low-oxygen environments (Lin et al., 2020). During the long-term natural evolution process, these bacteria have gained the ability to utilize the high iron and ROS contents in the environment (Guo et al., 2012; Li et al., 2017; Lin et al., 2020). The hypoxic microenvironment and the increased ROS levels in cells caused by high iron ion concentrations can trigger the magnetosome synthetic process (Lin et al., 2020). In this process, the gene encoding *mms6* plays a critical role (Tanaka et al., 2011; Staniland and Rawlings, 2016). This gene encodes a small molecular protein, MMS6, which only contains 61 amino acids, but has a strong ability to bind free irons. MMS6 possesses a ferrous ion binding site at C20, and the DDVED motif at the C-terminus is an iron ion binding site (Rawlings et al., 2016; Tunyasuvunakool et al., 2021). In addition, some studies have shown that *mms6* may be the key gene responsible for the utilization of ROS in magnetotactic archaea (Li et al., 2017). Therefore, this study utilized *mms6* to modify neural stem cells, so as to explore whether it was used by neural stem cells (NSCs) to reduce ROS in cells for resisting oxidative damage in the microenvironment.

## Materials and methods

### Generation and culture of midbrain neural stem cells

The study protocol was approved by the Institutional Animal Care and Use Committee of Shantou University. All procedures were conducted in adherence with the Guidelines for Animal Experimentation of Shantou University and the Chinese Guidelines for the Care and Use of Laboratory Animals. Twenty-four-hour Wistar neonatal rats were anesthetized with urethan and sterilized in 75% ethanol before the operation. After decapitation, the midbrain was dissected out on an ice board and transferred to a new dish with a culture medium. Their surface blood vessels were removed. The midbrain was sheared with microscopic scissors, and 5 ml of 0.05% trypsin (containing DNase 0.5 mg⋅L^–1^) was added and incubated at 37°C for 10 min. Trypsin digestion was terminated by adding a DMEM/F12 medium containing 1% FBS in an equal volume. The supernatant was discarded after centrifugation. The cells were dispersed by gentle repeated blowing. A serum-free culture medium of neural stem cells was added and transferred to 25 cm culture flasks and cultured in a thermostat. Neural stem cell culture medium: DMEM/F12 (1:1), 2% B27, bFGF (20 μg L^–1^), and 5% N_2_. The culture medium was refreshed every 7 days.

### Expression of *mms6* in neural stem cells

After codon optimization for mammalian expression, AMB-1 *mms6* from *Magnetospirillum magneticum* was cloned into the lentivirus vector (pHBLV-CMV-MCS -3FLAG-EF1-ZsGreen-T2A-PURO) and synthesized. To verify successful expression, both PCR and gene sequencing were performed. The *mms6* primers used for PCR were as follows: *mms6*-F, GGATCTATTTCCGGTGAATTCGCCACCATGGGATCCGC CACCATGCC; *mms6*-R, TAAGC- TTGGT ACCGAGGATCC AGCCAGAGCGTCCCTAAGTT.

To enhance the transfection efficiency, we cultured NSCs into single cells. Matrigel matrix glue was precoated on a six-well plate, placed in an incubator for at least 2 h, and then cleaned with PBS after drying. A serum-free neural stem cell culture medium was added. Well-growing suspensions of NSCs were transferred to a six-well plate for culturing. When NSCs were spread as single cells and their aggregation reached approximately 70%, lentivirus transfection was carried out. Before transfection, the original medium was removed, and 1 ml of fresh medium was added. Approximately 15 ml of the target virus was added to each well (an MOI of approximately 20:1) and incubated for 4 h. The supernatant was removed, and new medium was added. After 3 days, puromycin was used to screen untransfected cells (1 μg/ml), and the screening time was 3 days. The medium was refreshed after 3 days. Photographs were taken under a fluorescence microscope to determine the success rate of transfection.

### Cell proliferation test

Two types of NSCs (*mms6*-GFP-NSCs and GFP-NSCs) were cultured under different conditions: (1)0.5% CO_2_, 20% O_2_ and ferric citrate (0 μM); (2)0.5% CO_2_, 20% O_2_, and ferric citrate (320 μM); (3)0.5% CO_2_, 1% O_2_ and ferric citrate (0 μM); and (4)0.5% CO_2_, 1% O_2_, and ferric citrate (320 μM). To determine the effect of the different conditions on cell proliferation, we applied the CCK-8 test to detect the OD450 value of each cell culture via an enzyme labeling instrument. Two groups of cells were added to 96-well plates precoated with Matrigel gel (2 × 10^3^ cells/well, 200 μl serum-free NSC culture medium/well). Samples were photographed after 12 h, 1, 2, 4, and 8 days. Before the test, PBS was used to wash the plates, 100 μl of culture medium was added to each well, and 10 μl of CCK8 reagent was added to each well after 1 h. After incubation in the incubator for 2 h, the absorbance at 450 nm was measured by using a microplate reader.

### Mitochondrial superoxide production under the condition of oxidative damage induced by hypoxia or high levels of iron

To test the effect of *mms6* on mitochondrial superoxide production in a state of oxidative damage, we cultured NSCs under hypoxia or added a high concentration of ferric citrate (320 μM). To induce hypoxic conditions, we added 1.2 ml of paraffin oil to completely cover 6-well plates in which NSCs were cultured. After 24 h, both groups of NSCs were collected to detect mitochondrial superoxide by flow cytometry. The concentration of Mito-SOX (Thermo Fisher, M36008, USA) for testing was 5 μM, and the incubation time was 60 min. Excitation (nm) was at 488 and 580, and GFP as well as Mito-SOX signals were detected in the FL-1 and PE channels, respectively.

### Mitochondrial stress test

GFP-NSCs and *mms6*-GFP-NSCs were treated with/without ferric citrate (320 μM) for 2 weeks. These treated cells were then seeded in XFe 96-well microplates (6,000 cells/well) (Agilent Technologies, Santa Clara, CA, USA) overnight. Cells were washed and incubated in a base medium (Agilent Technologies) at 37°C for 1 h. The oxygen consumption rate (OCR) was measured in real time with a Mito Stress Test Kit using a Seahorse XFe96 Analyzer (Agilent Technologies) following the manufacturer’s instructions. Data were normalized by cell numbers that were measured by a YO-PRO^®^-1 Assay (Thermo Fisher Scientific).

### Transmission electron microscopy

Both NSC groups were cultured in a high concentration of ferric citrate for 1 week and were then collected for transmission electron microscopy (TEM) tests to detect the morphology of the mitochondria. The procedure was carried out as follows: Cells were digested, centrifuged, and fixed with glutaraldehyde. After rinsing and dehydration, acetone/epon812 was added for embedding, and cells were then baked in a 60°C oven for curing. After dressing the package, it was fixed on a slicing machine and sliced (layer thickness of 1 μm). The cell sections were placed on a 200-mesh φ 3 mm copper mesh to produce ultrathin sections (50–70 nm thick). The sections were selected with a lash pen, and sections were then taken with a steel ring. The selected sections were placed in a Petri dish, stained with a uranyl acetate solution for 30 min, and washed three times. After natural drying, lead citrate was added for dyeing and cleaning. The slices were dried and observed.

### Western blotting

To investigate the effects of *mms6* expression on the proteins associated with iron metabolism, we detected glutathione peroxidase 4 (GPX4; Abcam 125066, USA) and lipoxidase (LOX; Abcam 174316, USA) through Western blotting (WB). After the centrifugation of the collected cells, the protein concentration was measured. Aliquots of homogenate with equal protein concentrations (20 μg) were then added to SDS-PAGE gels (10%) and transferred to nitrocellulose membranes. After blocking in non-fat milk, the membranes were incubated overnight with primary antibodies (GPX4 1:1,000; LOX 1:000). The blots were then incubated with anti-rabbit IgG conjugated to IRDye for 1 h at room temperature. The film was then reacted with chemiluminescent detection reagents (reagent A:reagent B = 1:1) for 2 min. The film was then removed, excess liquid was shaken off, the PVDF film was wrapped with a preservative film, and an X film was used for photosensitivity, development, and fixation in a darkroom.

### Analog computation

We used Alphafold2 artificial intelligence protein simulation software based on MMseqs2 (Many-against-Many sequence searching) to predict the three-dimensional structure of the MMS6 protein, and evaluated the prediction model. Autodock docked Fe2+ with MMS6 and set the docked system with 2 positive charges and 3 positive charges, respectively. It was optimized by MOPAC to select sites with lower energy. PyMOL shows the docking conformation, and I-TASSER predicts the structure of GPX4. The conformation in the steady state was selected, and the structure of the model was evaluated by save6 V6; ZDOCK docked GPx4 proteins with the MMS6 protein after chelating iron ions; and PyMOL shows interaction.

### Statistics

The SPSS 12.0 software was used for the statistical analysis of the experimental data, and a *P*-value between groups of less than 0.05 was considered to be statistically significant. A non-parametric rank sum test and a two-sample *t*-test were used to compare differences between two groups. The comparision of growth rate among groups is performed by analyzing the differences in the five consecutive OD450 values using a method of repeated measure ANOVA. Differences in longitudinal changes in the survival rate between two groups were analyzed with repeated measures analysis of variance. Mauchly’s test of sphericity was used to check whether the variance in the difference between different measurements was equal in repeated measurements.

## Results

### Expression of *mms6* enhanced the tolerance of neural stem cells to iron overloading but not hypoxia

**FIGURE 1:**
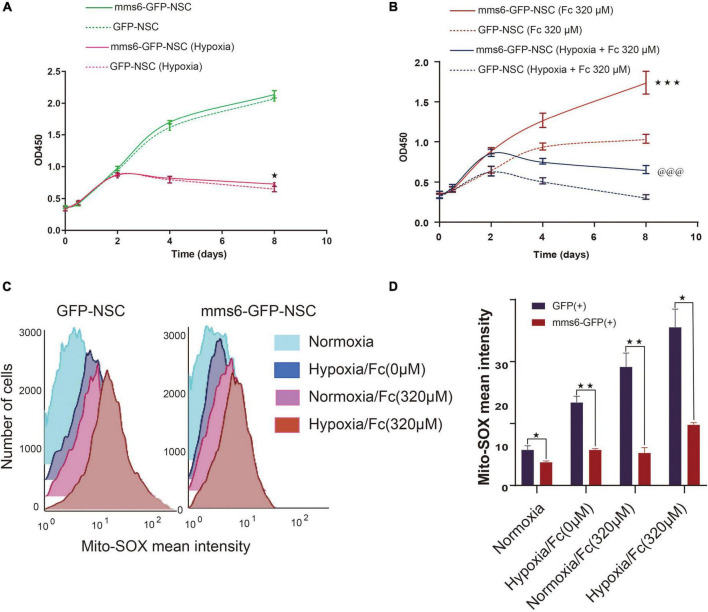
The expression of *mms6* can lead to the resistance of the harm caused by iron overloading on the growth of neural stem cells and decrease the production of superoxide anions. **(A)** Growth curve on consecutive days under normoxic/no iron and hypoxic/no iron conditions. (*) indicates that under hypoxic/no iron conditions, the OD450 value on the eighth day of *mms6*-GFP-NSCs was significantly higher than that of the control group (*P* < 0.05). **(B)** Growth curve on consecutive days under high levels of iron (320 μM) or hypoxia/high levels of iron (320 μM). *** or indicates a *P*-value of the significant difference of the 8-day growth curve between GFP-NSCs and *mms6*-GFP-NSCs, <0.001. **(C)** Histograms of superoxide anion staining of *mms6*-GFP-NSCs and GFP-NSCs cultured under different conditions. **(D)** Statistical graphs of the superoxide anion staining fluorescence intensity of *mms6*-GFP-positive NSCs and GFP-positive NSCs cultured under different conditions. * indicates a *P*-value <0.05; **<0.01; and ***<0.001.

Both iron overloading and hypoxia, following up with stroke, could induce oxidative damage and result in the death of stem cells. To test the effect of *mms6* on oxidative damage, we detected the survival rates of NSCs in these pathological states. Both *mms6*-GFP-NSCs and GFP-NSCs were cultured under four different conditions, namely normoxia/no iron, hypoxia/no iron, normoxia/high-level iron (320 μM), and hypoxia/high-level iron (320 μM). The OD450 value of each group was measured on the day 0, 1, 2, 4, and 8. The comparision of growth rate among groups is performed by analyzing the differences in the five consecutive OD450 values using a method of repeated measure ANOVA. As shown by the OD450 results in Figure 1D, *mms6* expression did not disturb the growth at the routine cell culture status (normoxic/no iron conditions). When we added a high level of iron in the culture medium, the growth rate of NSCs sharply decreased. *Mms6*-GFP-NSCs, but not those of the control group, exhibited tolerance to iron overloading. According to Figures 1B,D, *mms6*-GFP-NSCs showed a significantly increased survival rate in a high-iron environment regardless of whether it was normoxic (*F* = 85.98, *P* = 0.01). However, *mms6* expression did not significantly improve the growth rate under hypoxic conditions (*F* = 1.570, *P* = 0.530), although the OD450 value of *mms6*-GFP-NSCs was higher than that of GFP-NSCs on the 8th day (*t* = 2.945, *P* = 0.042) (Figures 1A,D). These data indicated that the expression of *mms6* in NSCs might contribute to the resistance of stem cells to iron overloading but not hypoxia.

### Expression of *mms6* decreased mitochondrial superoxide production and promoted mitochondrial function of neural stem cells in iron-loading conditions

Furthermore, this study examined whether the expression of *mms6* protected NSCs from oxidative damage. Mito-SOX is a sensitive indicator of changes in mitochondrial superoxide production. In this study, Mito-SOX fluorescence intensity was detected by flow cytometry to evaluate superoxide anion production under hypoxic and high-iron conditions. Typically, superoxide anion production was measured in *mms6*-GFP-NSCs and GPF-NSCs, respectively, under different culture conditions. As demonstrated in Figure 1C, when NSCs were cultured under both hypoxic and iron overloading conditions, their superoxide production increased significantly compared with those under normal conditions (Figure 1D). When compared with the control group, the expression of *mms6* significantly decreased their superoxide anion production. These results indicated that the expression of *mms6* might enhance the oxidation resistance of NSCs.

**FIGURE 2:**
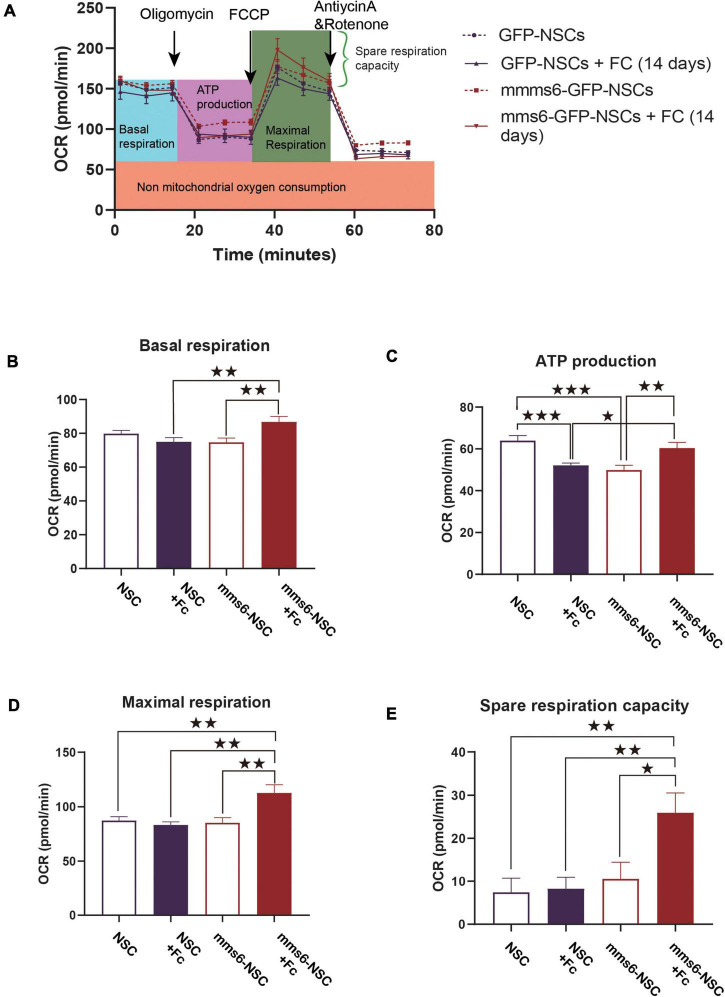
The expression of *mms6* helps stem cells resist the effect of iron overloading on mitochondrial respiratory functions. **(A)** Schematic diagram of the detection process of the oxygen consumption of cells in different groups; **(B)** basic oxygen consumption: the addition of exogenous iron increased the basal respiration capability of mms6-GFP-NSCs but not GFP-NSCs; **(C)** oxygen consumption during ATP production: the addition of exogenous iron decreased the ATP production of NSCs but increased it in *mms6*-GFP-NSCs; **(D)** the addition of exogenous iron enhanced the maximal oxygen consumption of *mms6*-GFP-NSCs but not that of the control group; and **(E)** the addition of exogenous iron obviously enhanced the spare oxygen consumption capacity of *mms6*-GFP-NSCs but not that of the control group. * indicates a *P*-value <0.05; **<0.01; and ***<0.001.

To test the effect on bioenergetic profiles, *mms6*-GFP-NSCs and GFP-NSCs were examined with a Seahorse XFe96 Analyzer (Figure 2A). In the absence of iron supplementation, *mms6*-GFP-NSCs had decreased ATP production (*t* = 4.265, *P* = 0.0003) but not basal OCR, maximal respiration, and spare respiration capacity (Figures 2B–E). When a high level of ferric citrate (320 μM) was added into the medium, ATP production decreased in GFP-NSCs compared with control NSCs (*t* = 4.49, *P* = 0.0002), whereas the opposite effect was observed in *mms6*-GFP-NSCs (*t* = 2.960, *P* = 0.0072; Figure 2C). At a high level of iron ions, *mms6*-GFP-NSCs exhibited higher basal OCR (*t* = 2.931, *P* = 0.0077; Figure 2B), maximal respiration (*t* = 3.665, *P* = 0.0014; Figure 2D), and spare respiration capacity (*t* = 3.336, *P* = 0.0030; Figure 2E) than those of GFP-NSCs. Collectively, our data indicated that the expression of *mms6* significantly promoted mitochondrial respiration and spare respiration capacity.

### *Mms6* increased the expression of glutathione peroxidase 4 and protected neural stem cells from mitochondrial damage induced by iron overloading

**FIGURE 3:**
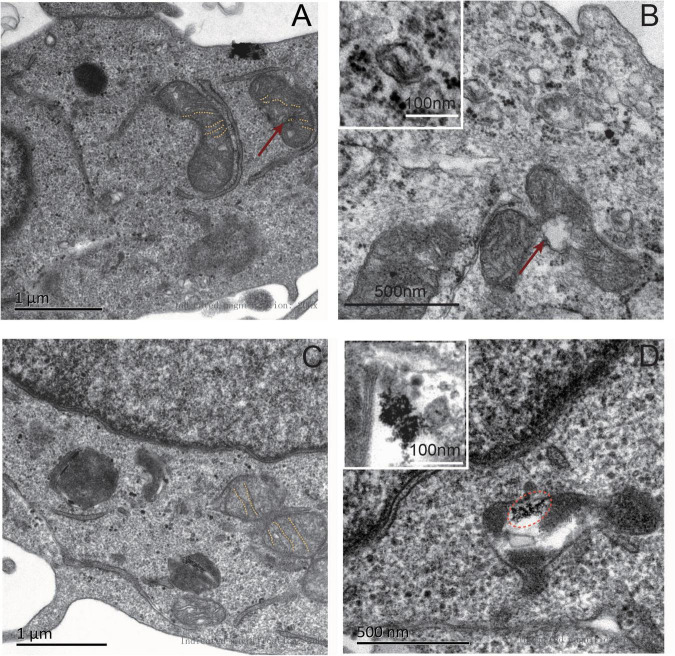
Transmission scanning electron microscopy (TEM) of NSCs under the condition of iron overloading (320 μM). **(A)** Under the condition of iron overload, a large number of iron granules were endocytosed in neural stem cells. A large amount of iron deposited in the cytoplasm or near the plasma membrane of GFP-NSCs. **(B)** Mitochondrial membrane structure of GFP-NSCs exhibited damaged (red arrow marked). **(C)** However, the mitochondria within with *mms6*-GPF-NSCs did not exhibit serious damage. **(D)** The iron particles in Mms6-GFP-NSCs could form a special crystalline in the cytoplasm [marked in the oval dotted box and displayed on the upper left panel of **(D)**].

Transmission electron microscopy was applied to observe the morphological changes in mitochondrial membrane structures. Under a high level of ferric citrate (320 μM), these iron particles were scattered on the cell membranes of GFP-NSCs, whereas iron particles in *mms6*-GFP-NSCs aggregated to form nanoparticles, as observed in Figures 3A,B. The mitochondrial membranes of GFP-NSCs without *mms6* expression were severely disrupted, along with mitochondrial damage, such as mitochondrial shrinkage (Figures 3C,D). However, the membrane structure of the mitochondrial crest in *mms6*-GFP-NSCs remained intact, with no obvious damage (Figures 3C,D).

**FIGURE 4:**
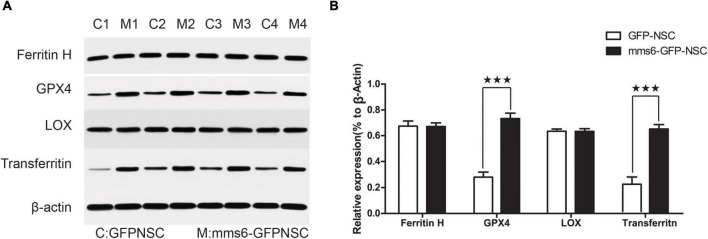
The expression of *mms6* increased the glutathione peroxidase 4 (GPX4) level of NSCs. **(A)** Western blotting (WB) of mms6-GFP-NSCs and GFP-NSCs; **(B)** the results of WB showed that the expression of GPX4, but not that of LOX, was significantly increased by the expression of *mms6*. MN, mms6-GFP-NSCs; GN, GFP-NSCs. *** indicates a *P*-value <0.001.

Iron loading may result in ferroptosis (Gomes et al., 2019). To further investigate the possible effect of mms6 on ferroptosis signaling, the expression of GPX4 and LOX was detected by WB assays (Figure 4A). GPX4 and LOX are the most important enzymes that regulate the iron-metabolism-related redox reactions (Bird et al., 2016), which are in a balanced state to regulate iron and oxygen metabolism (Bird et al., 2016). LOX is an oxidase containing non-heme proteins that mediates the oxidative reaction of iron ions in cells after overloading and produces a large number of hydroxyl radicals, whereas GPX4 is an important antioxidant enzyme in cells. Our data indicated that *mms6* did not increase the expression of LOX in cells (Figure 4B, *t* = 0.875, *P* = 0.958), but that it did increase that of GPX4 (Figure 4B, *t* = −7.916, *P* < 0.001), which also explained the reason why *mms6* had an antioxidant effect. Ferritin H and transferrin reflects intracellular iron metabolisms. We also detected the expression of Ferritin H and transferrin (Figure 2A). The results indicated *mms6* increased the expression of transferrin (*t* = −6.609, *P* = 0.001) but not Ferritin H (*t* = 0.066, *P* = 0.950).

### The MMS6 protein chelates ferrous iron through m6A and interacts with glutathione peroxidase 4

**FIGURE 5:**
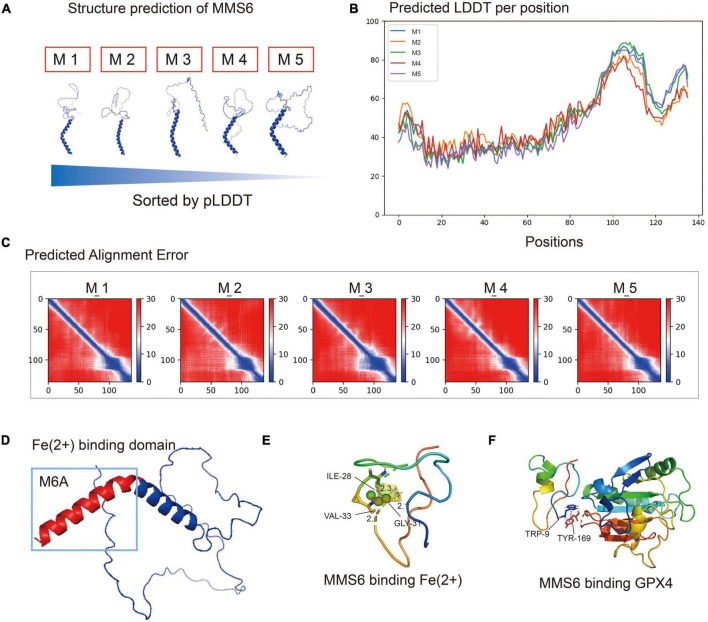
The MMS6 protein chelates ferrous iron through m6A and interacts with glutathione peroxidase 4 (GPX4). **(A)** The five protein structure models predicted by Alphafold2 are sorted according to the pLDDT (predicted local distance difference test). **(B)** Predicted local distance difference test (pLDDT) of each amino acid position in MMS6 protein structure models. **(C)** Predicted alignment error (PAE) values of five MMS6 protein structure models. **(D)** The chelating sites of MMS6 and Fe2+are distributed in the M6A sequence at the C-terminal. **(E)** The N-terminal after the chelation of MMS6 with Fe2+; the β-helices increase and the conformational stability increases. **(F)** The location where glutathione peroxidase 4 (GPX4) combines with MMS6 may be TRP-9 and TRY-169.

In order to determine the antioxidant mechanism, we analyzed the interaction of the MMS6 protein with ferrous ion and GPX4 via analog computation. We used AlphaFold2 artificial intelligence protein structure predictions based on MMseqs2 (Many-against-Many sequence searching) to predict the three-dimensional structure of the MMS6 protein (Seeliger and de Groot, 2010; Bird et al., 2016). As shown in Figures 5A–C, five structure prediction models were evaluated by alignment error and exhibited high quality. We then used PyMOL to mark the divalent iron binding region, M6A, of MMS6 (Figure 5D). After the chelation of the N-terminal of the MMS6 protein with Fe2^+^, the βhelix increased, and thus its conformational stability increased (Figure 5E). Additionally, we performed molecular docking (Seeliger and de Groot, 2010; Bird et al., 2016) to explore whether the MMS6 protein interacted with GPX4. The results showed that the docking score of the GPx4 and MMS6 docking complex was 978.573, and that the zrank energy was−151.374 kcal/mol (Figure 5F). The results indicated that there was the possibility of interaction between MMS6 and GPX4. This result provided a molecular mimicry basis for the subsequent physical and chemical study of the MMS6 protein.

## Discussion

In the present study, we first investigated whether the biomagnetic coding gene *mms6* had the potential ability to protect stem cells from oxidative damage in stroke. Our preliminary data exhibited exciting prospects of *mms6* in antioxidation in mammals. Inserting the magnetotactic bacteria gene *mms6* improved the capability of stem cells to survive in iron overloading conditions. Iron overloading is an inevitable state after stroke, especially in the late stage of a cerebral hemorrhage. A local iron overloading microenvironment would significantly weaken the therapeutic effect of stem cell transplantation. Our findings provided a novel path to solve such problems.

Stem cell transplantation is a promising strategy for the management of stroke and neurodegenerative diseases (Jiang et al., 2019; Sun and Alkon, 2019; Parmar et al., 2020). However, there is still a critical problem to be solved after transplantation, that is, the problem of pathological transplantation environments (Lisowski et al., 2018). When stem cells are transplanted into the brain, the conditions of high oxygen concentration and sufficient energy supply *in vitro* are transferred into the ischemic, hypoxic, and inflammatory microenvironments (Hou et al., 2017). Moreover, blood seepage and ischemia in the operation area will lead to an increase in iron in the stem cell microenvironment *in vitro*, along with increases in hydroxyl free radicals and ROS in hypoxic cells (Armand et al., 2012). These factors significantly reduce the survival rate of NSCs (De Filippis and Delia, 2011; Armand et al., 2012; Khacho et al., 2016; Lange et al., 2016; Khacho and Slack, 2017). Our team inserted a magnetosome mineralization gene into the genome of NSCs to investigate the effect of antioxidant activity. Our data provided primary evidence that the gene might be a new candidate antioxidant that improves the tolerance of stem cells in pathological microenvironments.

*mms6* is a critical gene of magnetotactic bacteria that is responsible for mineralization (Tanaka et al., 2011). It can guide the synthesis of ferromagnetic nanoparticles by crystallizing from free iron ions in cells. When mms6 is expressed in NSCs, it facilitates the utilization of free iron ions in cells and makes cells tolerant to hypoxic and high-iron environments (Guo et al., 2012; Lin et al., 2020). In hypoxic and high-iron-ion environments, long-term natural selection has endowed magnetotactic bacteria with the ability to resist low oxygen and high iron levels (Lin et al., 2020), as well as to make good use of magnetite. As a result, magnetotactic bacteria can utilize iron ions in microenvironments, transform them into energy, and synthesize ferromagnetic nanoparticles in cells (Johnsen and Lohmann, 2005; Lin et al., 2020). Therefore, magnetotactic bacteria are also used to scavenge harmful minerals from the environment in bioengineering and environmental engineering (Dieudonné et al., 2020). The addition of mms6 contributes to the ability of magnetotactic bacteria to scavenge intracellular free iron ions.

At the same time, our data confirmed the role of *mms6* expression in mitochondrial protection. The expression of mms6 enabled NSCs to resist damage caused by high iron ion levels and hypoxia in the microenvironment, and significantly reduced mitochondrial superoxide anion production, thereby weakening mitochondrial damage. The results were further confirmed by TEM. MMS6 binds to intracellular free iron ions and mobilizes intracellular iron activities. Our study found that *mms6* expression did not affect intracellular ferritin, but that it increased the expression of transferrin. This study confirmed that the synthesis of *mms6* in cells mobilized intracellular functional transferrin, and that mammalian cells effectively expressed and executed *mms6* function, even though the underlying mechanism remained unclear. *mms6*-bound iron ions did not trigger iron-death-related enzymes, but increased the expression of antioxidant enzymes. Our results showed that *mms6* expression did not increase the expression of LOX, but that it did increase that of GPX4. GPX4 and LOX are the most important enzymes that regulate the iron-metabolism-related redox reactions, which are in a balanced state to regulate iron and oxygen metabolism (Bogdan et al., 2016). We speculated that they might be related to the function of *mms6* itself. MMS6 combines with intracellular free iron ions, which significantly reduces the production of superoxide and hydroxyl radicals. Consequently, the downregulation of GPX4 enables cells to resist against the oxidative damage caused by iron-overloading.

We also found that the expression of *mms6* enhanced ATP production in NSCs under high-iron conditions. The results indicated that the expression of *mms6* significantly promoted mitochondrial respiration and spare respiration capacity. This was consistent with the TEM results *mms6*-modified NSCs experienced less mitochondrial membrane destruction under high-iron conditions. ATP production is associated with the generation of ROS, which are responsible for many negative feedback mechanisms of physiological reactions (Giniatullin et al., 2005; Sies and Jones, 2020). We speculated that the decrease in ATP production was not associated with C-terminal activity, which has been proven to show a strong binding ability to free iron. Fe2+ is not responsible for the decrease in ATP production, because it has been proven to have a negative relationship with ATP production. Additionally, there is no evidence proving the relationship between ATP production and Fe3+ levels. Collectively, this evidence suggested that the *mms6* gene did not interfere with the normal physiological activities of stem cells.

Considering all of the above results, it was concluded that the expression of *mms6* helped stem cells to reduce ROS levels in cells, thus facilitating resistance to the oxidative damage caused by the microenvironment. This discovery will provide good prospects for its application in NSC tracing *in vivo*, and also as a feasible strategy for enhancing the survival rate of stem cells. We believe that this result will pave the way to the application of *mms6* due to its antioxidant effect. This is also an important example that we have learned from magnetotactic bacteria, a kind of ancient bacteria.

## Data availability statement

The raw data supporting the conclusions of this article will be made available by the authors, without undue reservation.

## Ethics statement

The study protocol was approved by the Institutional Animal Care and Use Committee of Shantou University.

## Author contributions

J-HZ: conception, supervision, and design of this article. JC: supervision, funding, and editing the manuscript. X-YZ: supervision and editing the manuscript. N-LW: design of this article, cell experiments, and manuscript preparation. H-LT: experiments and manuscript preparation. WX: TEM and analog computation. QX: Seahorse experiments, data analysis, and editing the manuscript. YZ: manuscript editing and data analysis. All authors in the article have approved the submitted version.

## Abbreviations

NSC: neural stem cell
ROS: reactive oxygen species
GPX4: glutathione peroxidase 4
OCR: oxygen consumption rate
TEM: transmission electron microscopy
LOX: lipoxidase.
